# Medial Pterygoid Muscles Penetration by Tubero-Pterygoid Implants: Clinical, Anatomical and Statistical Insights Regarding Temporo-Mandibular Disorders (TMDs)

**DOI:** 10.3390/life16020350

**Published:** 2026-02-18

**Authors:** Łukasz Pałka, Vivek Gaur, Calin Fodor, Magdalena Gębska, Mehul Jani, Marta Bieńkowska, Bartosz Dalewski

**Affiliations:** 1 Private Dental Practice, Rzeszowska 2, 68-200 Żary, Poland; 2 Jaipur Dental College, Maharaj Vinayak Global University, Jaipur 302038, India; 3 Dr. Fodor Romulus Calin‘s Clinic of Dentistry and Implantology, Romania Str. Dragos Voda Nr. 8, 405300 Gherla, Romania; 4 Department of Rehabilitation Musculoskeletal System, Pomeranian Medical University, 70-204 Szczecin, Poland; 5 Department of Oral and Maxillofacial Surgery, College of Dental Sciences and Research Centre, Gujarat 382115, India; 6 REG-MED Dental Practice, Rzeszowska 2, 68-200 Żary, Poland; 7 Department of Dental Prosthetics, Pomeranian Medical University, 70-111 Szczecin, Poland

**Keywords:** medial pterygoid muscle, dental implants, maxillary rehabilitation, cone-beam computed tomography, temporomandibular joint disorders

## Abstract

Background: The medial pterygoid muscle (MPM) is frequently implicated in pain and dysfunction in patients with temporomandibular disorders (TMDs), owing to its functional complexity, susceptibility to overload, and rich neuromuscular control. Paradoxically, in patients rehabilitated with tubero-pterygoid implants, whose apices often penetrate or traverse the MPM attachment, no pain, trismus, or TMD-related symptoms are typically observed. Objective: The aim of this study was to evaluate the impact of implant penetration into the medial pterygoid muscle using CBCT and clinical examination after surgery and during follow-up visits. Methods: A retrospective observational study was conducted on 56 patients receiving a total of 116 tubero-pterygoid implants protruding beyond the pterygoid process of the sphenoid bone. Patients were divided into two groups according to implant penetration depth (<2 mm and >2 mm), with a minimum follow-up period of 12 months. Clinical outcomes related to pain, muscle disorders, and TMD symptoms were assessed. Results: Throughout the observation period, all patients remained free of pain, muscular disorders, and signs or symptoms of TMD, regardless of the degree of muscular penetration. Statistical analysis revealed no association between penetration depth and adverse clinical outcomes. Conclusions: The combined clinical and statistical evidence indicates that transmuscular penetration of the MPM by tubero-pterygoid implants is safe and well tolerated. These findings challenge traditional assumptions regarding MPM sensitivity and provide important guidance for surgical planning and maxillary rehabilitation strategies.

## 1. Introduction

The medial pterygoid muscle is the one of the four paired muscles responsible for chewing. It typically consists of two heads, the deep and superficial. The deeper head arises from the medial surface of the lateral pterygoid plate and from the pyramidal process of the palatine bone when the superficial head arises from the pyramidal process and from the tuberosity of the maxilla and together ends at the medial surface of the mandible near its angle [1,2].

Nonetheless, in the literature, there is some inconsistency in descriptions of the origin of the deep head of the muscle [3,4,5]. With the development of reconstructive and rehabilitative dentistry, the pterygoid muscles have become an area of interest for several specialties, including temporomandibular joint (TMJ) disorders, dental prosthetics, traumatology, and, more recently, implantology—particularly with the increasing use of tubero-pterygoid anchorage [6,7,8,9,10,11]. Although its clinical importance suggests that a proper understanding of their function is beneficial, both the physiological and anatomical descriptions of these muscles are sometimes contradictory. On the one hand, it is sensitive to injury, tension, and pressure, often serving as an indicator of TMJ disorders. On the other hand, in implantology, where tubero-pterygoid implants are used, the implant apex frequently penetrates the muscle’s attachment site between the pterygoid processes of the sphenoid bone without causing pain or affecting the function of the stomatognathic system.

Where do these discrepancies come from? Clinical studies show that only 4.0% of needle insertions into the muscle tissue elicit pain [12]. In contrast, during dry needling of the medial pterygoid muscle, four out of five patients report pain [13]. Moreover, unlike a needle, an implant has a diameter that is several dozen times larger, is permanently placed within the muscle, may have a rough surface or threads, and chronically irritates the tissue—yet clinical incidents of pain from such implants are extremely rare.

To explain this phenomenon, we must consider the motor and sensory innervation of the medial pterygoid muscle, as well as the number and distribution of muscle spindles, which are sensory receptors that detect muscle stretch and may also contain pain receptors [14,15,16].

Currently, cone-beam computed tomography (CBCT) is the gold standard for imaging after implant placement and during follow-up visits. However, it provides limited soft tissue resolution for structures such as muscles. While less effective than MRI for visualizing muscle tissue, CBCT can still be used to assess muscle changes, often with the aid of specialized software [17,18,19].

The aim of this study was to evaluate the impact of implant penetration into the medial pterygoid muscle using CBCT and clinical examination after surgery and during follow-up visits.

This study was conducted and reported in accordance with the STROBE guidelines for observational studies.

## 2. Materials and Methods

### 2.1. Study Design and Setting

This retrospective observational study analyzed CBCT scans and clinical outcomes collected between January 2023 and December 2025 from dental implantology centers in Poland, Romania, and India. The study period refers to the dates of CBCT acquisition and clinical follow-up documentation available in the archives.

### 2.2. Participants and Inclusion Criteria

The study followed methodological standards established in earlier research by the same scientific group [20,21]. Inclusion criteria were the presence of tubero-pterygoid implants (single-piece implants with smooth surface) and a follow-up period of at least 12 months. Exclusion criteria were incomplete visualization of the tubero-pterygoid region, no implant protrusion beyond the bone plates, not attending control visits and follow-up shorter than 12 months. When multiple CBCT scans were available for the same patient, only the scan corresponding to the qualifying follow-up period was included. The study size reflected the number of eligible cases available in the archives during the study period.

### 2.3. Imaging and Measurements

The dataset included 68 CBCT scans of the posterior maxilla region with 135 tubero-pterygoid implants, retrospectively selected from patient archives. Inclusion was limited to cases with complete visualization of the tubero-pterygoid region and implant penetration of the bone plates by at least 1 mm. Participants were not actively recruited, and CBCT images were anonymized prior to analysis, retaining only demographic data (age and gender). Therefore, patient consent was not required, as the scans had been originally acquired for clinical purposes unrelated to this study. No identifying information was accessible to the researchers at any point during the selection or analysis process.

Assessment and measurement of the region of interest (ROI) were independently performed by two experienced implantologists. Clinical outcomes assessed during follow-up included muscle pain, limited mouth opening, and signs or symptoms of temporomandibular disorders. Beforementioned symptoms were identified based on documentation from routine clinical examinations and follow-up visit records, without the use of standardized diagnostic scales. Radiological outcomes assessed on CBCT included osteolysis around the implant apex region, and implant mobility was assessed clinically during follow-up visits. These evaluations were conducted on axial and sagittal radiological planes using specialized imaging software (ver. 1.0.6.0.1, Ez3D-i; Vatech, Hwaseong-si, Gyeonggi-do, Republic of Korea). To verify intra- and inter-observer reliability, a subset of scans was randomly re-evaluated after several weeks. Additionally, two independent researchers reviewed the images to confirm measurement accuracy and consistency. Discrepancies were resolved through discussion and consensus.

Clinical follow-up data were extracted from routine postoperative and follow-up visit records at each participating center.

### 2.4. Statistical Analysis

Chi-square tests, Student’s *t*-test, the Mann–Whitney U test, Spearman’s correlation analysis, and binomial tests were used for statistical evaluation. Statistical significance was set at *p* < 0.05. Penetration depth was analyzed using a predefined categorical cut-off of 2 mm (<2 mm vs. >2 mm). Analyses were performed at the patient level and implant level as reported in the Results, using standard statistical software (ver. 13.3.721, Statistica, Statsof, Kraków, Poland). Subgroup analyses were performed according to sex and penetration depth categories. There were no missing data for age, sex, penetration depth, or clinical outcome variables in the included cases.

### 2.5. Ethics Statement

The study was conducted in accordance with the Declaration of Helsinki, and ethical review and approval were waived due to its retrospective character (Opinion of the Chairman of the Bioethics Committee at the District Medical Chamber in Zielona Góra No. 03/179/2025, dated 18 June 2025).

### 2.6. Bias

To reduce measurement bias, ROI measurements were performed independently by two experienced implantologists, with repeat assessment of a subset of scans and consensus resolution of discrepancies. Selection bias is possible due to retrospective archive sampling; therefore, all cases meeting inclusion criteria within the study period were considered.

## 3. Results

A total of 68 CBCT scans with 135 tubero-pterygoid implants were initially identified from archives. After applying eligibility criteria, 56 patients with 116 implants were included in the final analysis.

### 3.1. Demographic Characteristics of the Study Group

#### Population Structure

**Figure 1:**
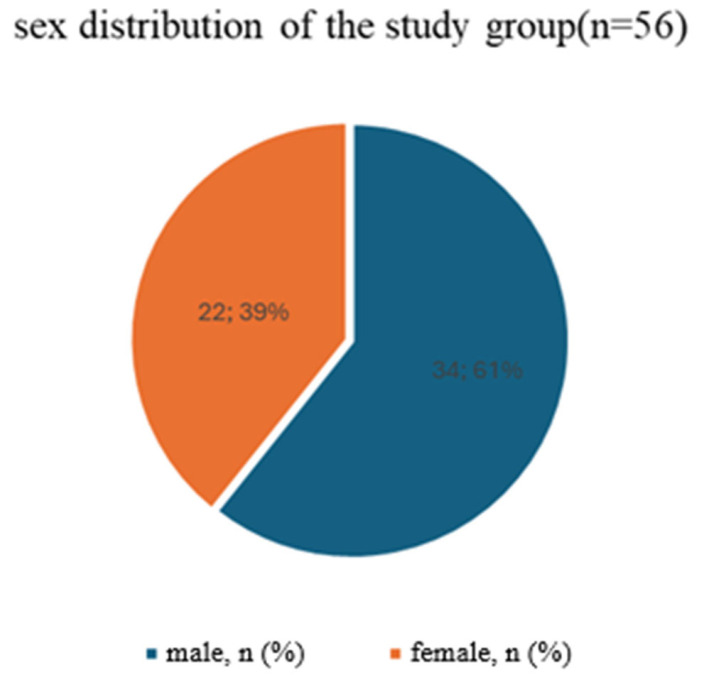
Sex distribution of the study group.

The study group consisted of 56 patients (34 men and 22 women) with a total of 116 tubero-pterygoid implants (Figure 1). The mean age of the patients was 63.68 ± 10.92 years (range: 18–80 years). Although men constituted the majority (60.7%), a chi-square test showed no statistically significant difference in sex proportions (χ^2^ = 2.5714, *p* = 0.1088).

The median age was 65 years.

**Figure 2:**
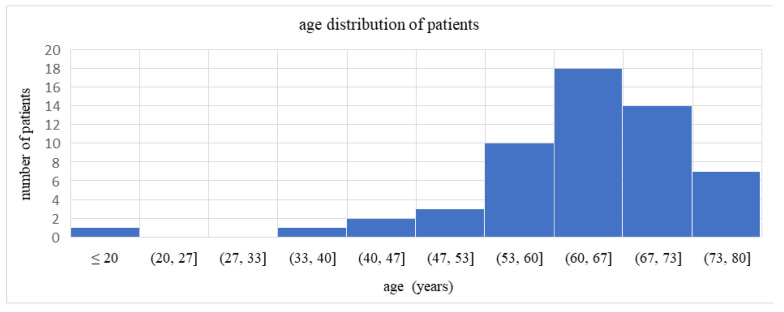
Age distribution of patients in the study group.

A comparison of mean age between men (63.12 ± 12.66 years) and women (64.55 ± 7.68 years) showed no statistically significant difference (Figure 2). Student’s *t*-test (*t* = −0.4746, *p* = 0.6370) clearly indicated no significant age difference between sexes.

### 3.2. Distribution of Penetration Depth

**Figure 3:**
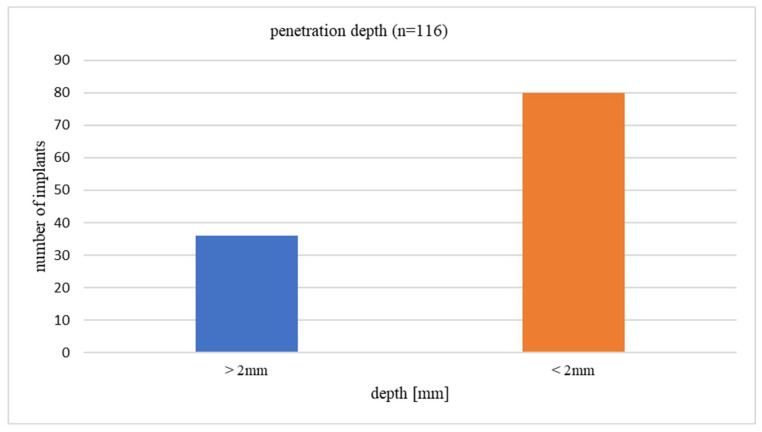
Distribution of penetration depth of the tubero-pterygoid implants into the MPM.

Of the 116 implants, 69.0% (*n* = 80) protruded beyond the pterygoid process of the sphenoid bone, thus penetrating medial pterygoid muscle (MPM) to a depth of <2 mm, while 31.0% (*n* = 36) penetrated deeper than 2 mm (Figure 3).

At the patient level, 46.4% (*n* = 26) had at least one implant penetrating the muscle > 2 mm, whereas 53.6% (*n* = 30) had all implants penetrating < 2 mm. The mean number of implants per patient was 2.07 ± 1.37, with the most common configurations being one implant (*n* = 33) or four implants (*n* = 17). This distribution reflects the varied bone conditions of patients, from single tubero-pterygoid implants to double pterygoid approach.

**Figure 4:**
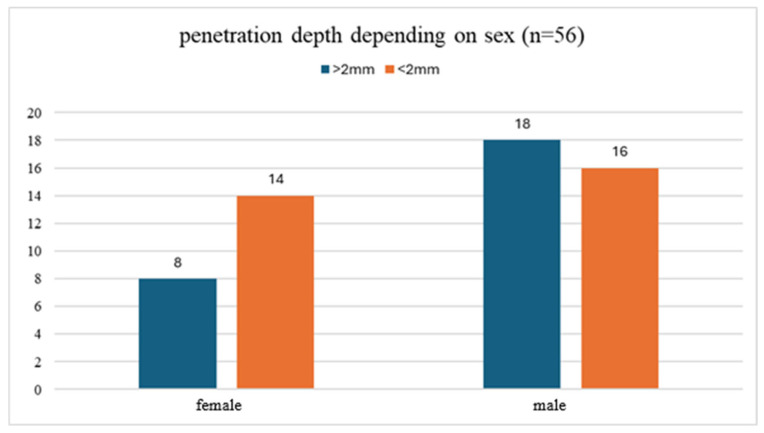
Comparison of implant penetration depth between sexes.

A chi-square test of independence showed no statistically significant association between patient sex and implant penetration depth (χ^2^ = 0.8846, *p* = 0.3470) as shown in Figure 4.

The Mann–Whitney U test demonstrated no significant age differences based on penetration depth (U = 336.5, *p* = 0.3832). Patients with >2 mm penetration had a mean age of 62.81 ± 10.06 years, compared with 64.43 ± 11.73 years for those with <2 mm penetration.

Spearman’s correlation between patient age and number of implants was ρ = 0.0099 (*p* = 0.9421).

**Figure 5:**
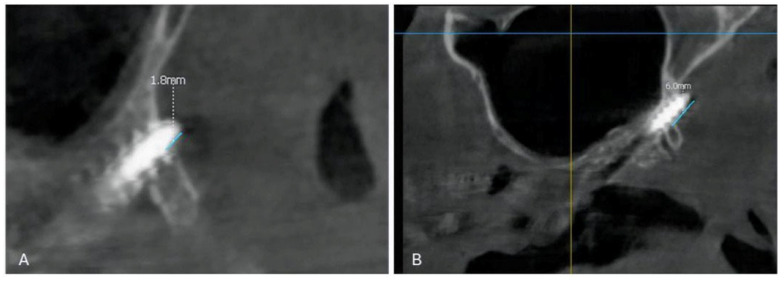
Representative CBCT images of tubero-pterygoid implant penetration into the medial pterygoid muscle region. (**A**) Sagittal CBCT view demonstrating limited penetration of a tubero-pterygoid implant beyond the pterygoid process of the sphenoid bone <2 mm. (**B**) Sagittal CBCT view from a different patient illustrating deeper implant penetration into the tubero-pterygoid region >2 mm.

Representative CBCT images illustrating tubero-pterygoid implant penetration into the medial pterygoid muscle region are shown in Figure 5.

### 3.3. Clinical Outcomes

None of the 56 patients exhibited radiological osteolysis, implant mobility, pain, limited mouth opening, or temporomandibular disorder symptoms during the 12-month follow-up period.

## 4. Discussion

The medial pterygoid muscle (MPM) presents a unique paradox in maxillofacial practice. It is often implicated as a source of pain in patients with temporomandibular disorders (TMDs), yet, paradoxically, in patients with pterygoid or tubero-pterygoid implants—where the implant apex is positioned directly within or adjacent to the muscle’s insertion—pain or dysfunction is typically not observed. Understanding this contrast requires consideration of neuromuscular physiology, local inflammatory responses, and the biomechanical context of both conditions.

The aim of this study was to evaluate the impact of implant penetration into the medial pterygoid muscle using CBCT and clinical examination after surgery and during follow-up visits. The absence of complications such as radiological osteolysis, implant mobility, pain, trismus, and TMD symptoms after 12 months of follow-up confirms that the study objectives were achieved.

The analysis of 56 patients with 116 tubero-pterygoid implants provides strong evidence supporting the clinical safety of this implant technique.

In patients with TMD, particularly those with myofascial pain syndrome, pain associated with the MPM is primarily of myogenous origin, linked to excessive loading, parafunctional habits, and central sensitization of the trigeminal nociceptive pathways [22,23]. The MPM’s dense motor innervation via the mandibular division of the trigeminal nerve (V3) and its relatively rich in spindles in the middle part of the muscle but and few or no spindles near the origins of the superficial and deep heads or near their insertion [15]. This distribution may correspond to the localization of myofascial trigger points specified as a hyperirritable spot that arises from muscles and their connective tissue [24]. Furthermore, electromyographic studies have demonstrated increased resting tonus and spontaneous activity in symptomatic patients, supporting the hypothesis of neuromuscular over-excitation rather than structural damage [25].

By contrast, the physiological environment of the MPM surrounding a properly positioned pterygoid implant is markedly different. The implant apex traverses the pterygoid process of the sphenoid bone and often engages the attachment zone of the head of the MPM. However, this penetration does not elicit pain for several reasons. First, the osseofixation converts the implant-bone interface into a stable, immobile unit, eliminating micro-motion that would otherwise activate nociceptors [26]. Secondly, the chronic adaptation of surrounding muscle fibers leads to a form of ‘functional desensitization’ due to neural accommodation and the absence of inflammatory mediators typical of overuse injuries [27]. Histological evidence from animal models suggests that once the implant surface becomes integrated with mineralized tissue, the adjacent soft tissue undergoes fibrous reorganization, reducing the density of free nerve endings [28].

Another important factor is the mechanical context. In TMD, pain is driven by repeated, dynamic contraction against resistance or disc displacement, whereas the presence of a fixated implant represents a static, non-contractile stimulus. The implant does not produce tensile or shear stress but instead establishes a new mechanical equilibrium within the muscle’s attachment zone. Moreover, MPM’s proprioceptive control depends primarily on central modulation rather than continuous peripheral feedback; thus, once an implant is integrated and non-inflammatory, it ceases to provide nociceptive input [29,30].

Finally, the absence of pain in pterygoid implants may also reflect differences in the microenvironment of neural endings. The neuromuscular spindles present in medial pterygoid muscles are usually located in deep areas of this muscle [31], and its innervation is primarily motor [32]. Once the local tissue adapts to the foreign body, the muscle fibers reorganize around the implant, encapsulated by fibrous connective tissue with reduced vascularity and neural density [33]. Therefore, while acute surgical trauma might transiently stimulate nociceptors, long-term stability and lack of micromotion prevent chronic pain.

In conclusion, the absence of pain in patients with single-piece, smooth-surface pterygoid implants, despite their transmuscular placement, likely results from the combination of mechanical stability, absence of dynamic overload, neural adaptation, and fibrous encapsulation. In contrast, TMD-related MPM pain arises from continuous hyperactivity, inflammatory microtrauma, and altered central pain modulation. This understanding highlights the distinct pathophysiological mechanisms underlying the same anatomical structure under different clinical conditions.

From a statistical perspective, the most relevant finding of this study is the uniform absence of adverse clinical and radiological outcomes across all evaluated parameters during the follow-up period. The consistency of complication-free outcomes in all patients and implants suggests that these findings are unlikely to be attributable to random variation and instead reflect a stable and reproducible clinical pattern. The absence of adverse events precluded the application of multivariate statistical models for risk-factor analysis; however, this limitation arises directly from the uniformly positive outcomes observed and simultaneously indicates a robust short-term safety profile for tubero-pterygoid implants.

Clinical and Research Implications

The absence of complications despite the penetration of the sensitive medial pterygoid muscle confirms the clinical paradox described in the literature: mechanically stable implants do not elicit pathological symptoms. The lack of association between penetration depth, patient age, sex, or number of implants and the occurrence of complications suggests that this technique can be safely applied across a wide range of maxillary rehabilitation cases.

These findings support the hypothesis of neural adaptation and soft-tissue reorganization around stable implants, which differentiates this scenario from the dynamic muscle overload observed in temporomandibular disorders. This has important implications for surgical planning and patient qualification in advanced maxillary rehabilitation.

## 5. Conclusions

This study draws the following conclusions:

1. Tubero-pterygoid implant penetration into the medial pterygoid muscle is safe and well-tolerated.
2. Neither shallow (<2 mm) nor deep (>2 mm) penetration produced pain, trismus, osteolysis, or TMD symptoms.
3. Age, sex, and number of implants did not influence outcomes.
4. Mechanical stability, neural adaptation, and fibrous tissue reorganization may explain the absence of adverse symptoms.

## Data Availability

Data are available from the corresponding author upon reasonable request.

## References

[1] Fonseca R.J., Walker R.V., Barber H.D., Powers M.P., Frost D.E. (2013). Oral and Maxillofacial Trauma.

[2] Hannam A.G., McMillan A.S. (1994). Internal organization in the human jaw muscles. Crit. Rev. Oral Biol. Med..

[3] Sakamoto Y., Akita K. (2004). Spatial relationships between masticatory muscles and their innervating nerves in man with special reference to the medial pterygoid muscle and its accessory muscle bundle. Surg. Radiol. Anat..

[4] El Haddioui A., Bravetti P., Gaudy J.F. (2007). Anatomical study of the arrangement and attachments of the human medial pterygoid muscle. Surg. Radiol. Anat..

[5] Baker E.W., Schuenke M., Schulte E., Schumacher U. (2010). Head and Neck Anatomy for Dental Medicine.

[6] Bakke M. (1993). Mandibular elevator muscles: Physiology, action, and effect of dental occlusion. Scand. J. Dent. Res..

[7] Bakke M., Möller E. (1992). Craniomandibular disorders and masticatory muscle function. Scand. J. Dent. Res..

[8] Choi T.H., Kim B.I., Chung C.J., Kim H.J., Baik H.S., Park Y.C., Lee K.J. (2015). Assessment of masticatory function in patients with non-sagittal occlusal discrepancies. J. Oral Rehabil..

[9] Murray G.M., Carignan C., Whittle T., Gal J.A., Best C. (2022). Pterygoid muscle activity in speech: A preliminary investigation. J. Oral Rehabil..

[10] Bidra A.S., Peña-Cardelles J.F., Iverson M. (2023). Implants in the pterygoid region: An updated systematic review of modern roughened surface implants. J. Prosthodont..

[11] Konstantinović V.S., Abd-Ul-Salam H., Jelovac D., Ivanjac F., Miličić B. (2023). Pterygoid and tuberosity implants in the atrophic posterior maxilla: A retrospective cohort study. J. Prosthet. Dent..

[12] Partanen J.V., Lajunen H., Liljander S.K. (2023). Muscle spindles as pain receptors. BMJ Neurol. Open.

[13] Mesa-Jiménez J.A., Fernández-de-Las-Peñas C., Koppenhaver S.L., Sánchez-Gutiérrez J., Arias-Buría J.L. (2020). Cadaveric and in vivo validation of needle placement in the medial pterygoid muscle. Musculoskelet. Sci. Pract..

[14] Kröger S., Watkins B. (2021). Muscle spindle function in healthy and diseased muscle. Skelet. Muscle.

[15] Bhojwani V., Ghabriel M.N., Mihailidis S., Townsend G.C. (2017). The human medial pterygoid muscle: Attachments and distribution of muscle spindles. Clin. Anat..

[16] Akcora D.S., Karacan E., Yapicier O., Ogut E., Barut C. (2025). Evaluation of muscle spindle density and distribution of certain mimic muscles: A cadaveric study. Bratisl. Med. J..

[17] Greenberg A.M. (2015). Cone beam computed tomography scanning and diagnosis for dental implants. Oral Maxillofac. Surg. Clin. N. Am..

[18] Shen Y., Li X., Feng X., Zhang C., Shang Y., Lin J. (2025). The effects of masseter muscle morphology on three-dimensional occlusion and temporomandibular joint in adult patients with skeletal class II malocclusion: A CBCT study. BMC Oral Health.

[19] Öncü E., Kuzey N., Baltaş E., Çağlayan F. (2025). CBCT-based measurement of lateral pterygoid muscle length in TMJ dysfunction. BMC Oral Health.

[20] Calin F., Dalewski B., Ellmann M., Kiczmer P., Ihde S., Bieńkowska M., Kotuła J., Pałka Ł. (2025). CBCT evaluation of maxillary incisive canal characteristics among population in regard to possibility of implant cortical anchorage—A multicenter study. Dent. J..

[21] Calin F.R., Dalewski B., Ihde S., Czuczwał M., Konstantinovic V.S., Gaur V., Kotuła J., Pałka Ł. (2025). CBCT evaluation of cortical bone thickness in the nasal floor and lateral wall: Considerations for implant anchorage—A retrospective multicentre study. Dent. J..

[22] Travell J.G., Simons D.G. (1999). Myofascial Pain and Dysfunction: The Trigger Point Manual.

[23] Türker K.S., Jenkins M. (2000). The motor control of jaw movements: Current perspectives. J. Oral Rehabil..

[24] Fernández-de-Las-Peñas C., Nijs J. (2019). Trigger point dry needling for the treatment of myofascial pain syndrome: Current perspectives within a pain neuroscience paradigm. J. Pain Res..

[25] Rodrigues Conti P.C., Ferreira P.M., Pegoraro L.F., Conti J.V. (2001). Effect of parafunctional habits on temporomandibular disorders. J. Oral Rehabil..

[26] Ihde S., Pałka Ł., Ihde A. (2017). Functional load transmission of pterygoid implants: A biomechanical analysis. Int. J. Oral Implantol. Clin. Res..

[27] Wada S., Koizumi H., Maeda T. (2004). Adaptation of orofacial muscle afferents following chronic mechanical stimulation. Neurosci. Res..

[28] Schliephake H., Aref A., Scharnweber D. (2005). Bone formation and soft tissue response in the vicinity of titanium implants. Clin. Oral Implants Res..

[29] Türker K.S. (2002). Reflex control of human jaw muscles. Crit. Rev. Oral Biol. Med..

[30] Sessle B.J. (2011). Peripheral and central mechanisms of orofacial pain and their clinical correlates. J. Oral Rehabil..

[31] Bazan E., Issa J.P., Watanabe I.S., Mandarim-de-Lacerda C.A., Del Bel E.A., Iyomasa M.M. (2008). Ultrastructural and biochemical changes of the medial pterygoid muscle induced by unilateral exodontia. Micron.

[32] Akita K., Sakaguchi-Kuma T., Fukino K., Ono T. (2019). Masticatory muscles and branches of mandibular nerve: Positional relationships between various muscle bundles and their innervating branches. Anat. Rec..

[33] Steed M.B., Al-Shebely M., Leake D. (2021). Histological response of soft tissue around pterygoid implants: A preliminary report. Int. J. Oral Maxillofac. Surg..

